# Golden Gait: An Optimization Theory Perspective on Human and Humanoid Walking

**DOI:** 10.3389/fnbot.2017.00069

**Published:** 2017-12-19

**Authors:** Marco Iosa, Giovanni Morone, Stefano Paolucci

**Affiliations:** Clinical Laboratory of Experimental Neurorehabilitation, IRCCS Fondazione Santa Lucia, Rome, Italy

**Keywords:** walking, neuroscience, anthropometry, rehabilitation, divine proportion, golden section, fractal, game theory

## Abstract

Human walking is a complex task which includes hundreds of muscles, bones and joints working together to deliver harmonic movements with the need of finding equilibrium between moving forward and maintaining stability. Many different computational approaches have been used to explain human walking mechanisms, from pendular model to fractal approaches. A new perspective can be gained from using the principles developed in the field of Optimization theory and in particularly the branch of Game Theory. In particular we provide a new insight into human walking showing as the trade-off between advancement and equilibrium managed during walking has the same solution of the Ultimatum game, one of the most famous paradigms of game theory, and this solution is the golden ratio. The golden ratio is an irrational number that was found in many biological and natural systems self-organized in a harmonic, asymmetric, and fractal structure. Recently, the golden ratio has also been found as the equilibrium point between two players involved into the Ultimatum Game. It has been suggested that this result can be due to the fact that the golden ratio is perceived as the fairest asymmetric solution by the two players. The golden ratio is also the most common proportion between stance and swing phase of human walking. This approach may explain the importance of harmony in human walking, and provide new perspectives for developing quantitative assessment of human walking, efficient humanoid robotic walkers, and effective neurorobots for rehabilitation.

## Introduction

Optimization theory is a branch of mathematics aiming at identifying the best choice, from some set of available alternatives, that optimizes (maximizes or minimizes) a specific target function (Asghar Bhatti, 2000). A well-known application of optimization theory to human locomotion refers to the fact that the comfortable walking speed is, for healthy individuals, that minimizing the energy consumption (Ralston, 1958; Miller et al., 2012; Oh et al., 2012; Long and Srinivasan, 2013; Seethapathi and Srinivasan, 2015).

Game Theory is a branch of Optimization Theory in which there is not just one function to optimize, but there is the need to identify the best compromise among some entities involved into the problem (Kolokoltsov and Malafeyev, 2010). Game Theory has become a large and powerful theoretical framework providing mathematical models for predicting the choices of rational entities (usually called players) in conflict or in cooperation tasks (Rapoport, 1974; Sanfey, 2007). Mainly used in psychology, economy, political science, logic, computer science, Game Theory has also been enlarged to biology (Maynard Smith, 1982). Following this approach, game theoretical methods have been used in biochemistry and biophysics (Schuster et al., 2008), with some studies considering cells (Gatenby and Vincent, 2003) and even molecules (Bohl et al., 2004) as “players” working together or being in competition for the same objective.

The idea of applying game theory to human walking proposed in this article originates by the observation that current advances in these so different research fields reported the same solution for two apparently different problems. In fact, the same equilibrium point was found in human walking and Ultimatum game: this point coincides with the so-called golden ratio.

The golden ratio (φ) is the solution of the problem already reported by Euclid in III century B.C. to cut a given straight line so that the proportion between the shorter part to the longer one is the same as the longer part to the whole. It is an irrational number already found in many physical, biological fractal structures that are self-organized so that the larger-scale structure resembles the subunit structure (King et al., 2004; Yamagishi and Shimabukuro, 2008). In fact, it was found in structures of animal bodies (Livio, 2003) and plants’ leaves (Okabe, 2011), in the solar systems (Lombardi and Lombardi, 1984), replicated in architecture (Hemenway, 2005) and in certain musical rhythms (Garland, 1995), as well as in financial market patterns (Agaian and Gill, 2017). In humans, harmonic proportions have been found in the physiological activity of the heart (Yetkin et al., 2013) and in anthropometry (Davis and Altevogt, 1979), as depicted in figurative arts (Hemenway, 2005). In general, the golden ratio has been found as the best choice for many biological processes (Bartl et al., 2010; Yetkin et al., 2013; Schuster et al., 2017).

## Game Theory: The Ultimatum Game Equilibrium

The Ultimatum game is a model game developed for analyzing fairness, and it is one of the most famous paradigms of Game Theory. In this game, two players must divide a sum of money: the proposer has to specify this division and the responder has the option of accepting or rejecting the offer (Sanfey, 2007). If the offer is accepted, the sum is divided as proposed, otherwise neither player receives anything. A rational intelligence should accept any offer and, knowing this, the proposer could offer the smallest nonzero amount (this choice is called Nash equilibrium). However, most of responders reject small proposals because judged unfair. Another point of equilibrium predicted by game theory is an offer split in 50% and 50% proportion (equipartition).

But most of the studies reported that the two solutions reported above are not the most common, and the average offer ranges around 62% for player 1 and 38% for player 2 (Oosterbeek et al., 2004; Henrich and Silk, 2013).

Some recent researchers (Schuster, 2017; Suleiman, 2017) noted that this division, chosen by subjects as the fairest one, is very close to the value of the golden ratio, and it was due to the fact that responders may tend to accept an amount that is in a proportion with that taken by proposer when this proportion is the same between the latter part remaining at the proposer with respect to the whole amount.

**Figure 1:**
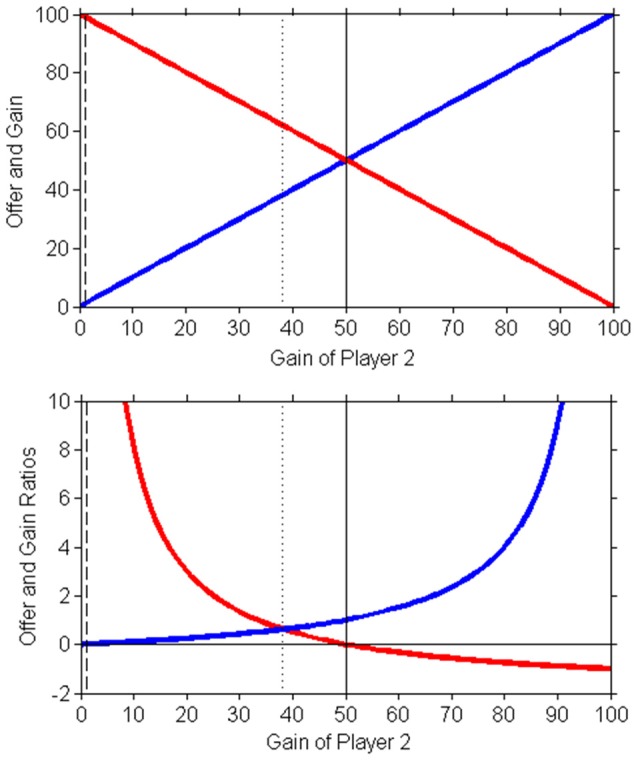
Schematic representation of the Ultimatum Game of Game Theory. Above the amount taken by player 1 (red line) and that taken by player 2 (blue line). The dash line is the Nash equilibrium (chosen by completely rational intelligence that accepts each offer higher than zero), the solid line the equipartition (fifty-fifty), the dot line the golden ratio solution (golden equilibrium, that observed in experimental data). Below, the offer and gain are expressed as proportions: red line is the offer ratio (expressed in percentage of whole amount) and the blue line is the gain of player 2 (with respect to that taken by player 1). The equilibrium is given by golden ratio solution (partition: about 38.2% for player 2 and 61.8% for player 1): player 2 tends to accept an offer of the minor fraction of φ (38.2%) because they accept a proportion of that taken by player 1 if this latter sum is in the same proportion with the whole amount.

Figure 1 theoretically depicts the Ultimatum Game. This “equality of fractions” is conceivably perceived by both players as the fairest asymmetric division (Schuster, 2017).

The Golden Ratio plays a role also in other games analyzed in game theory (Camerer et al., 2004; Berg et al., 2015) and in the so-called justice evaluation function (Jasso, 2007). The Ultimatum Game is even used in economics (Güth and van Damme, 1998), and as its solution, the golden ratio has been reported as an example of “Economic Harmony” (Suleiman, 2017).

## Current Advances in Human Walking: The Golden Gait

Many different computational approaches have been used to explain human walking mechanisms, from inverted pendulum model (Ivanenko et al., 2007) to fractal approaches (Hausdorff et al., 1995).

**Figure 2:**
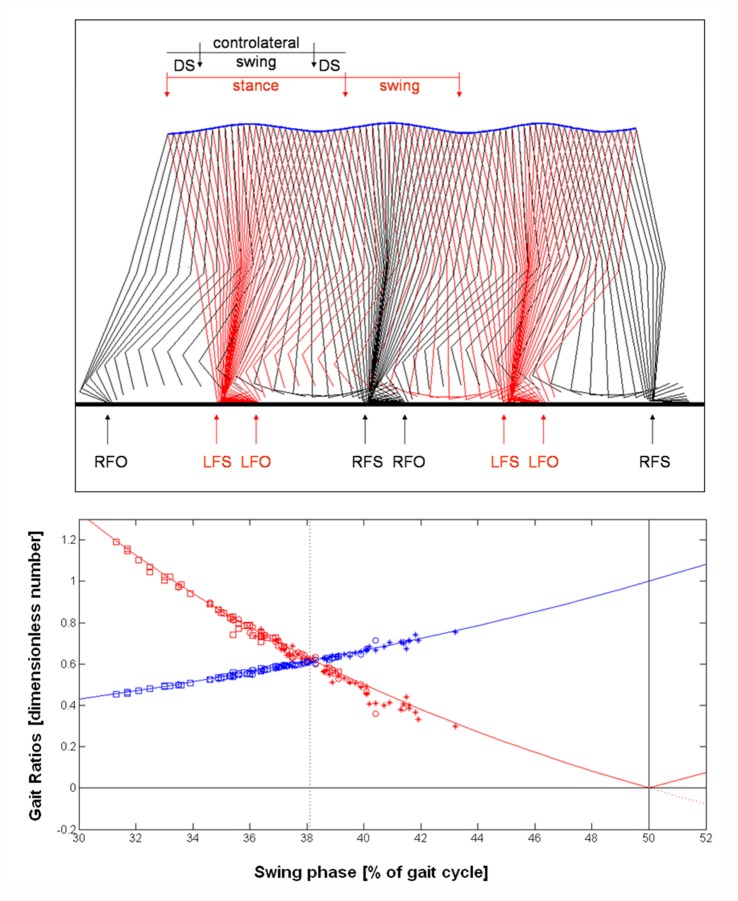
Above: stick diagram of walking obtained linking right (R, black lines) and left (L, red lines) markers of foot toe and ankle (foot), ankle and knee (thigh), knee and center of mass (lengthened shank). The blue line is the trajectory of the whole body center of mass. FS, foot strike; FO, foot off; DS, Double Support. Stereophotogrammetric data of an healthy subject walking at comfortable speed (Iosa et al., 2007) were used for depicting this stick diagram. Below: Theoretical model of the gait ratios DS/Sw (red line) and Sw/St (blue line). Absolute value of Ds/Sw was adopted for Sw > 50%, being this value the transition limit between walking and running. Experimental data of slow (squares), comfortable (circles) and fast (stars) walking are also shown. Data related to comfortable walking converged to the Golden Equilibrium.

The inverted pendulum model refers to the pendular trajectory of center of mass and to the relevant transfer from potential to kinetic energy and its reverse (Ivanenko et al., 2007; McGrath et al., 2015). This trajectory can be seen in the above plot of Figure 2 showing the stick diagram, a typical figure formed linking anatomical points and obtained using stereophotogrammetric systems (Ivanenko et al., 2007). At comfortable speed (about 1.4 m/s), locomotor system saves energy by exchanging forward kinetic energy and gravitational potential energy of the center of mass during the inverted-pendulum oscillation of stance (Cavagna and Margaria, 1966), and by ballistically oscillating the limb as a compound-pendulum during swing (Mochon and McMahon, 1980). Thus muscle activity is only required to oppose gravity, maintain postural configurations in the face of interaction torques, and reintegrate energy losses during each cycle (Lacquaniti et al., 2012). Inverted pendulum is a simplified model in which a rigid rod represents the leg during stance phase, other more sophisticated models take into account also the stiffness and the elastic properties of the lower limbs (Lipfert et al., 2012).

The fractal approach refers to the idea that gait has a fractal structure, id est the larger-scale structure resembles the subunit structure (Hausdorff et al., 1995). The simplest examples of fractals are the structures based on the golden ratio. Recently, the Golden ratio has been found in gait cycle (GC) as the proportion between the stance duration (the longer part of a stride) and the swing duration (the shorter part). When the stance and swing are in the golden ratio, gait phases revealed a fractal structure based on the property of autosimilarity, with the same proportionality emerging between units and consecutive subunits of gait, resembling in each part the same whole structure (Iosa et al., 2016d) as follows:

$$\phi =\text{ ​​}\frac{\text{1+}\sqrt{\text{5}}}{\text{2}}=\frac{\text{swing}}{\text{double support}}=\frac{\text{stance}}{\text{swing}}\text{​​ }=\text{ ​​}\frac{\text{stride}}{\text{stance}}=\frac{\text{stride + stance}}{\text{stride}}=\frac{\text{2strides + stance}}{\text{stride + stance}}=\ldots$$

With the expression “golden gait” we refer to a gait in which the above equations are respected. Recent researches showed as golden gait can be a bridge between pendular model and fractal approach of walking (Iosa et al., 2016b). This relationship is based on the strict intertwine between structure and functioning, been the anthropometric proportion between the stature and the distance of center of mass from the ground close to the golden ratio (Davis and Altevogt, 1979), as also represented in the ancient Greeks’ sculptures (Di Dio et al., 2007).

However, the golden gait is not only related to the biomechanics of human body and its anthropometric proportions. In fact, the pendulum mechanism of walking should be considered as a forced oscillator for the need of rhythmic intervention of muscle activity and hence of nervous system (West and Scafetta, 2003). It has been hypothesized that a neural network formed by cerebellum, globus pallidum and central pattern generators of spinal cord is the generator of the golden ratio harmonic rhythm of walking (Iosa et al., 2016d). This hypothesis can be supported by clinical data showing as the golden ratio based rhythm is altered in patients with cerebellar ataxia (Serrao et al., 2017) and Parkinson’s Disease (Iosa et al., 2016d), but not directly in presence of a damage of cortical areas due to stroke (Iosa et al., 2016a).

## Applying Game Theory to Human Walking

During walking there is only one subject, the walking person, but he/she has to manage between the need of advancing and the need of maintaining stability without falling.

The relationships between the gait phases, expressed in percentage of the whole GC (100%), can be written as follows:

$$\begin{array}{l} \text{St}=100-\text{Sw}\\ \text{DS}=\text{St}-\text{Sw}\prime \end{array}$$

where St = stance phase; Sw = swing phase; Sw′ = controlateral swing phase; DS = double support phase. For a symmetric walking Sw = Sw′ and hence:

DS=St−Sw=100−2Sw

The advancement depends on the speed of walking, the efficiency of walking optimized around the speed self-selected by subjects as the comfortable one (Cotes and Meade, 1960). Walking speed is linearly proportional to swing duration (Hebenstreit et al., 2015). So the advancement depends on the ratio swing/stance (Sw/St), with the limit of Sw/St < 1, otherwise (Sw > St) walking becomes running, with the absence of any double support phase (Mann and Hagy, 1980).

Gait stability has been warranted by the fact that stance phase is longer than swing phase, that generates the presence of a double support phase (DS), with both feet on the ground (Perry, 1992). In fact, the maintenance of equilibrium is due to a dynamic stability in which the fall of center of mass has been controlled by the swinging leg arriving to touch the ground before subject falls (Mrozowski et al., 2007). This stability can be increased by prolonged double support phase (Perry, 1992). Hence, upright stability is strictly related to the duration of the double support (DS) with respect to swing duration (DS/Sw).

Given the above equations, it is possible to report the two ratios DS/Sw and Sw/St (related to stability and advancement, respectively) as function of the only swing phase (Sw) as follows:

$$\{\begin{array}{c} \frac{\text{DS}}{\text{Sw}}=\frac{100-2\;\cdot \text{ Sw}}{\text{Sw}}\\ \frac{\text{Sw}}{\text{St}}=\frac{\text{Sw}}{100-\text{Sw}}\; \end{array}$$

These ratios can be seen as analogous to the offer of player 1 and the gain of player 2, respectively. Below plot of Figure 2 shows the curves described by these two functions. This approach (as well as this plot) clearly replicates that reported for gain and offer ratios of the Ultimatum Game curves (Figure 1).

In the range of swing phase values in which DS/Sw > Sw/St, the stability is favored over advancement. On the contrary, if DS/Sw < Sw/St, the advancement is favored over stability.

In this subplot of Figure 2 we also superimposed to the curves the experimental data extracted by a previous study on walking in healthy adults (Iosa et al., 2016a) and recorded using an optoelectronic system during comfortable walking (for details see Iosa et al., 2016a). Data recorded at slow and fast walking were not reported in that study, but collected in the same experimental session and reported here as original data that agree with those of literature (Perry, 1992).

Similarly to the responses observed for the Ultimatum Game, experimental data converged to this Golden equilibrium between advancement and stability.

Schuster (2017) suggested also an alternative approach for explaining the golden solution as the convergent equilibrium of the Ultimatum Game. This alternative way of explanation in terms of bargaining corresponds with the convergence of the following continued fraction that is another property of the golden ratio:

$$\varphi =1+\;\frac{1}{1+\;\frac{1}{1+\frac{1}{1+\ldots }}}$$

The results of this progressive division are: 3/2, 5/3, 8/5… All these numbers (1, 2, 3, 5, 8…) are consecutive Fibonacci numbers and the limit of the ratio between two consecutive Fibonacci numbers is the golden ratio.

Also this approach can be applied to human walking. In fact, the two limits are fixed by static posture (maximum stability, no advancement) and the limit of transition between walking and running (Sw = St = 50%). The following schema could be hence derived applying Fibonacci’s numbers (1, 2, 3, 5, 8) to walking, using the ratio between the gait cycle (GC) and the stance (St):

$$\begin{array}{l} \text{​}\frac{\text{GC}}{\text{St}}=1+\frac{1}{1+\frac{1}{1+\frac{1}{1+\ldots }}}\\ \text{Posture:}\frac{\text{GC}}{\text{St}}=1\to (stance=100\%)\\ \text{Transition to Running:}\frac{\text{GC}}{\text{St}}=1+1=2\;\to \text{(stance = 50\%)}\\ \text{Slow walking:}\frac{\text{GC}}{\text{St}}=1+\frac{1}{2}=\frac{3}{2}\to (\text{stance}=66.6\%)\\ \text{Fast walking:}\frac{\text{GC}}{\text{St}}=1+\frac{1}{1+\frac{1}{2}}=\frac{5}{3}\to (\text{stance}=60\%)\\ \text{Comfortable walking:}\frac{\text{GC}}{\text{St}}=1+\frac{1}{1+\frac{1}{1+\frac{1}{2}}}=\frac{8}{5}≅\varphi \to (\text{stance}=62.5\%) \end{array}$$

The foot-off occurring at the minima located at 5/8 of the GC approximates the golden ratio with a difference of only 0.7% of stride duration.

The most important similarity between Ultimatum Game and the stance/swing trade-off in GC is that both need an asymmetric equilibrium point. In fact, in the Ultimatum Game, subjects usually assume that the proposer has some priority because he has the whole amount and he is allowed to choose the offer (Ichinose, 2012). Similarly, walking cycle is characterized by the presence of a double support phase, existing only if St > Sw.

## Future Directions: From Hominids to Humanoid Walking, Passing through Robot-Assisted Walking

The experience of million of years of evolution between extreme solutions (posture and running) in search for optimal bipedal walking, seems to have achieved the golden compromise between stability and advancement, muscle work and efficiency (Massaad et al., 2007). This is confirmed by studies on patients, showing as the golden gait minimizes the energy cost optimizing the transfer between potential and kinetic energy (Serrao et al., 2017), and on virtual model of walking, tested in different initial conditions, always converging to the golden ratio as an equilibrium point (Dzelaidini et al., 2014). Surprisingly, even chimpanzee during bipedal walking showed a proportion between stance and stride that is about 0.65 (Demes et al., 2015), so that the inverse proportion is close to the golden ratio. It has been even hypothesized that humans evolved from hominids in the actual antrophometric dimensions in golden proportion for favoring this harmonic golden gait (Iosa et al., 2016b).

It is well known that patients with neurological impairments have an alteration of the percentage duration of stance and swing (Perry, 1992), but only recently this alteration was put in relationship to a deviation from the golden ratio (Iosa et al., 2016a, d; Serrao et al., 2017).

For years, it has been suggested that rehabilitation should drive walking patterns of individuals with a gait impairment at resembling the patterns of healthy individuals as closely as possible. However more recent results revealed a more complex scenario. Hak et al. (2014) showed that step length asymmetry in individuals with a transtibial amputation are functional in terms of stability: a training aiming at recovering gait symmetry may affect dynamic balance during gait in these subjects, as theoretically suggested earlier by Merker et al. (2011). Our perspective study can contribute in clarifying that the optimization should take into account more target functions at the same time, as in Game Theory, not just one. Future research should focus on a more in-depth understanding of how gait impairments influence human locomotion and which target functions should be trained for a better recovery of gait autonomy finding a favorable trade-off that could even be different from physiological one.

A clear example is that of humanoid robots, in which the choice of bioengineers is guided by the cost of these robots. For most of humanoid robots, walking is quite different from that of humans because the distance between the center of mass and ground is usually maintained constant (Massaad et al., 2007). These humanoid robots use sophisticated motor control to walk smoothly while demonstrating appalling inefficiency with excessive energy cost: in this case the management between stability and advancement has been achieved favoring the former one for reducing the risk of fall and hence of damages. In humans, a walk with a flat trajectory of center of mass need muscles working in unfavorable conditions, wasting energy (Massaad et al., 2007). From this point of view, passive-dynamic mechanical walkers, moving up and down their center of mass, rival with humans in terms of efficiency (Collins et al., 2005). Recently, a bipedal robot has been developed using a golden ratio algorithm for reproducing golden gait obtaining a harmonious walking patterns similar to those of humans (Tez and Kuşçu, 2017).

Robots for assisting human walking during rehabilitation have already been made for replicating a ratio between stance and swing close to the golden ratio: 60% and 40% (Hesse et al., 2000) or 62% and 38% (Volpini et al., 2017). However, this proportion was chosen just according to the principle of driving patients’ gait patterns resembling the physiological patterns. It is worthy to investigate whether the golden ratio can be applied in robotic-aided therapy not just because it is a physiological pattern, but because it is the best harmonious trade-off between stability and advancement. The golden gait, in fact, seems to be a key of efficiency for locomotor control and stimulating the sensori-motor system at the frequency of the golden ratio may facilitate the restoring of the harmonic activity of neural circuits at the basis of walking.

Neurorobots already have the potential for accurately assessing motor functioning, therapy progresses and for providing patients with stimulation and real-time feedback on movement performance (Iosa et al., 2016c). This approach should take into account the golden ratio, using this proportion for stimulating and quantitatively assessing gait harmony (Torricelli et al., 2014; Saner et al., 2017), as suggested for assessing electrocardiographic patterns with respect to the deviation from the golden ratio of the cardiac cycle ratios (Ciucurel et al., 2018).

## Conclusion

Despite the golden ratio has often been reported as a “magic number”, it is just the solution of a simple problem of a geometrical problem already reported by Euclid in III century B.C. and then also found as solution adopted by many biological and physical systems, including fractal structures and human physiology (Iosa, 2016). It has also been judged as the fairest proportion for geometrical figures (Green, 1995) and anthropomorphic sculptures (Di Dio et al., 2007) in psychological studies.

In the Game Theory, the golden ratio was found as the best equilibrium for the Ultimatum Game because judged as the fairest solution by the players. Following a similar approach, we showed that the golden ratio is also the best solution for managing advancement and stability during human walking optimizing bipedal efficiency.

As Suleiman (2014) noted and Schuster (2017) reported, the word “fair” has a double meaning, that of equitable and beautiful. For human walking, golden gait is also the most efficient. Once again, we can learn a lesson from Ancient Greek for whom a beautiful harmonic structure is strictly intertwined with an efficient virtuous functioning.

## Ethics Statement

This perspective article include some data already published (and here only re-analyzed) in Iosa et al. (2016b).

## Author Contributions

MI developed the idea and the application of game theory to human walking. GM critically reviewed the original manuscript. SP coordinated and supervisioned the writing process. All the authors contributed to the discussion and the development of article structure.

## Conflict of Interest Statement

The authors declare that the research was conducted in the absence of any commercial or financial relationships that could be construed as a potential conflict of interest.
